# Gene Expression Analysis of the Effect of Ischemic Infarction in Whole Blood

**DOI:** 10.3390/ijms18112335

**Published:** 2017-11-05

**Authors:** Ayako Takuma, Arata Abe, Yoshikazu Saito, Chikako Nito, Masayuki Ueda, Yoshiro Ishimaru, Hideki Harada, Keiko Abe, Kazumi Kimura, Tomiko Asakura

**Affiliations:** 1 Department of Applied Biological Chemistry, Graduate School of Agricultural and Life Sciences, The University of Tokyo, Bunkyo-ku, Tokyo 113-8657, Japan; ayako.tkm712@gmail.com (A.T.); yx554833@rd6.so-net.ne.jp (Y.S.); yishimaru@meiji.ac.jp (Y.I.); aka7308@mail.ecc.u-tokyo.ac.jp (K.A.); 2 Department of Neurological Science, Graduate School of Medicine, Nippon Medical School, Bunkyo-ku, Tokyo 113-8602, Japan; abe@nms.ac.jp (A.A.); cnito@nms.ac.jp (C.N.); k-kimura@nms.ac.jp (K.K.); 3 Department of Neurology and Stroke Medicine, Tokyo Metropolitan Tama Medical Center, Fuchu, Tokyo 183-8524, Japan; ueda@nms.ac.jp; 4 Department of Anesthesiology, Kurume University School of Medicine, Kurume, Fukuoka 830-0011, Japan; hidehara@med.kurume-u.ac.jp

**Keywords:** stroke, middle cerebral artery occlusion model, gene expression analysis, messenger RNA, microRNA, rat

## Abstract

Given the abundance of stroke patients and deaths from stroke worldwide, many studies concerning the aftermath of stroke are being carried out. To reveal the precise effect of ischemic infarction, we conducted a comprehensive gene expression analysis. Alongside a middle cerebral artery occlusion (MCAO) Sprague–Dawley rat model, we used a group undergoing sham surgery for comparison, which was the same as MCAO surgery but without blood vessel occlusion. Subsequently, infarction of the brains of MCAO-treated rats occurred, but did not occur in the sham-treated rats. Using whole blood, we carried out DNA microarray analysis, revealing the gene expression alterations caused by stroke. Downregulation of immune pathways and cluster of differentiation (CD) molecules indicated immunodepression. By conducting miRNA microarray analysis, we extracted seven miRNAs as significantly regulated: miR-107-5p, miR-383-5p, miR-24-1-5p, mir-191b, miR-196b-5p, and miR-3552 were upregulated, and mir-194-1 was downregulated. Among these seven miRNAs, three had one target mRNA each that was extracted as differentially expressed, and the expression levels of all pairs were inversely correlated. This indicates the occurrence of miRNA–mRNA regulatory systems in blood: between miR-107-5p and H2A histone family member Z (H2afz), miR-196b-5p and protein tyrosine phosphatase receptor type C (Ptprc), and miR-3552 and serine/arginine-rich splicing factor 2 (Srsf2). Moreover, six miRNAs had matching human miRNAs with similar sequences, which are potential human stroke biomarkers.

## 1. Introduction

Stroke is a major cause of death worldwide, and the numbers of affected patients and associated deaths have been rising. Moreover, these numbers are expected to continue to rise [1]. Against this background, many studies concerning the aftermath of stroke are being conducted.

One major topic of such studies is immunology. Specifically, many experimental and clinical studies have investigated the immune system to determine the pathophysiological changes that occur after stroke [2,3]. It is known that, while an inflammatory response occurs in the brain after stroke, the whole body suffers from an immunodepressed state. Another important topic in this field is the search for biomarkers. Many studies involving the search for biomarkers that enable diagnosis, differentiation between stroke types, and prediction of the reoccurrence of stroke are being undertaken [4]. In addition, some recent studies investigating miRNAs as biomarkers for stroke have been reported [5,6,7,8].

In such studies, the middle cerebral artery occlusion (MCAO) model has been widely utilized [9,10,11]. However, in many of these studies, the MCAO-treated group was compared with a control group that underwent either no surgery at all or sham surgery that only involved cutting open the skin. To overcome this problem and create a more realistic control group, in our study, along with the MCAO model rats, we used sham-operated rats into which we inserted uncoated thread into the blood vessel to imitate the procedure used on the MCAO-operated rats. This enabled us to observe the effect of ischemic infarction more precisely, since any difference in effect between the two groups of undergoing an operation was eliminated.

In this study, we conducted a comprehensive gene expression analysis to investigate the alterations in gene expression after stroke. Upon applying the MCAO and sham methods described above, the difference in gene expression between the two groups could be determined to reflect only the phenomenon of cerebral ischemic infarction, thus providing new insights. We used whole blood and first performed DNA microarray analysis to investigate the comprehensive change in gene expression after stroke. We then analyzed the miRNA in blood using a microarray and determined the correlations between mRNA and miRNA. In the pursuit of new biomarker candidates, we also identified human miRNAs that correspond to the miRNA biomarker candidates.

## 2. Results

### 2.1. Internal Condition of the Brain after Stroke

To investigate the internal condition of the brain of MCAO- and sham-operated rats, 2,3,5-triphenyltetrazolium chloride (TTC) staining was performed. A total of six slices from the ventral side were obtained and subjected to TTC staining. Using the image analysis software ImageJ, total volume (mm^3^), nonedema volume (mm^3^), edema volume (mm^3^), infarct volume (mm^3^), edema index (%), and infarct index (%) for each sample were calculated. For the experiment from this point, three samples of MCAO group were selected by the criterion of infarct index (17–21%), which resulted in *n* = 3 for each group.

**Figure 1:**
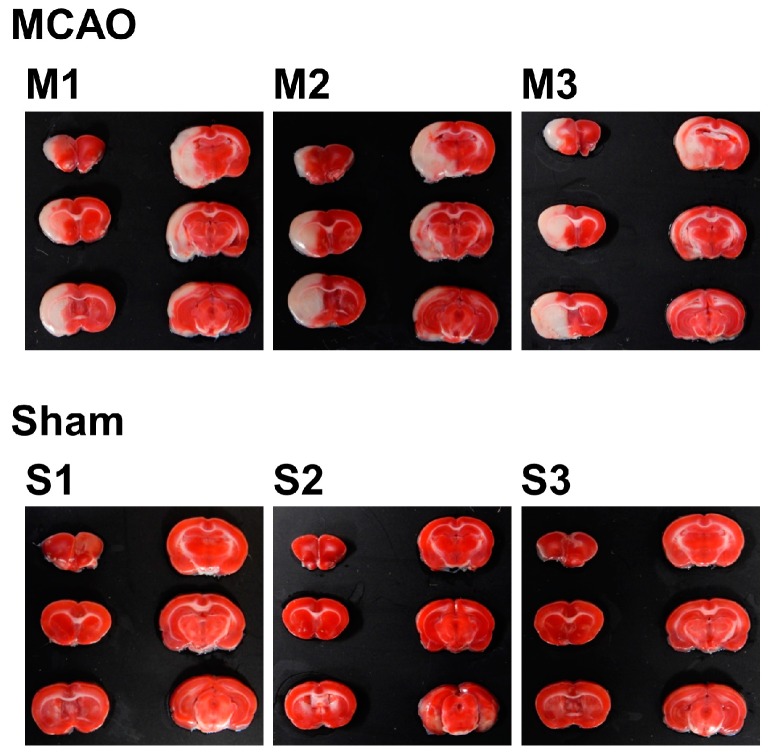
2,3,5-triphenyltetrazolium chloride (TTC)-stained brain slices of MCAO- or sham-treated rats. The slices were taken from the ventral side and lined up from upper left to bottom right, with the dorsal surface up. The infarcted area is shown as the white part.

For the selected samples, TTC-stained brain slice samples are shown in Figure 1 lined up with the dorsal surface up, from upper left to bottom right. Since TTC stains only living cells, the infarcted areas are shown in white. With the origin of the left MCA occluded, most of the left hemisphere, starting from the striatum to the cortex, was infarcted in the MCAO group. In contrast, sham-operated rats showed no signs of infarction.

The result of image analysis is shown in Table 1. The mean infarct index was 18.94% in the MCAO group, while there was no infarct in the sham group. The mean edema index was 29.08% in the MCAO group, while for the sham group, it was 6.98%, which clearly shows the effect of swelling of the infarct area. These figures show the effect of occlusion in the MCAO group, which cannot be seen in the sham group.

### 2.2. mRNA Expression in Blood

**Figure 2:**
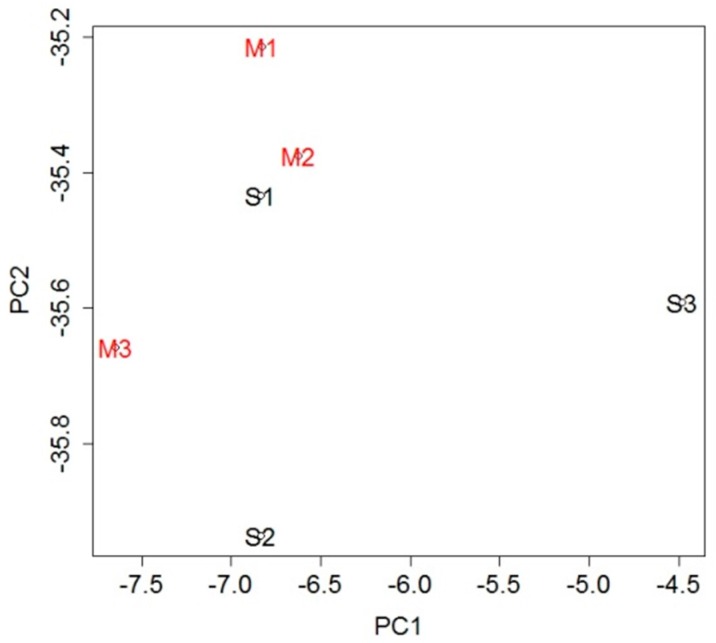
Principal component analysis (PCA) using expression levels of all probe sets. The general clustering of the samples within each group indicates the difference between the groups.

To investigate the genetic changes caused by stroke, gene expression in the blood was analyzed and compared between the MCAO and sham groups. A total of 232 probe sets (Table S1), which met the criterion of false discovery rate (FDR) <0.05, were extracted as differentially expressed genes (DEGs): 42 probe sets were upregulated in the MCAO group and 190 were downregulated when compared with the sham group. To evaluate overall gene expression, we performed principal component analysis (PCA). There was general clustering of the samples within each group, indicating the difference between them (Figure 2).

To examine the DEGs further, we performed canonical pathway analysis and listed the pathways regulated by the DEGs. Pathways with *p*-value < 0.05 and |*Z*-score| ≥ 2 were extracted as being significantly regulated (Table 2). Among these pathways, immune-related ones were significantly downregulated, such as CD28 signaling in T helper cells, role of NFAT in regulation of the immune response, fMLP signaling in neutrophils, CXCR4 signaling, Fcγ receptor-mediated phagocytosis in macrophages and monocytes, and IL-8 signaling.

Since the canonical pathway analysis showed the downregulation of immune-related pathways in the MCAO group, we investigated immune-related genes among the DEGs. We found that immune-related CD molecules such as CD36, CD3g, CD47, CD59, and CD74 were downregulated (Table 3).

### 2.3. miRNA Expression

Since miRNAs circulate in the body through the blood vessels, they regulate gene expression in the cells of various tissues. We thought that changes in mRNA expression in blood may be regulated by miRNAs originating in the brain and circulating in the blood. Therefore, we analyzed and compared the expression of miRNAs in the blood between the MCAO and sham groups. By using Affymetrix Transcriptome Analysis Console (TAC) software, we found seven miRNAs that were significantly regulated and that fulfilled the applied criteria (ANOVA *p*-value < 0.05, |fold change| ≥ 1.5) (Table 4). Six of them, namely, rno-miR-107-5p, rno-miR-383-5p, rno-miR-24-1-5p, rno-mir-191b, rno-miR-196b-5p, and rno-miR-3552, were upregulated, while only rno-mir-194-1 was downregulated in the MCAO group compared with the sham group.

To examine the changes caused by the miRNAs, target genes of the mature miRNAs were searched for in the database miRDB. Among the target genes of the significantly regulated mature miRNAs, there were three mRNAs that were in common with the DEGs extracted by DNA microarray analysis of the blood sample (Table S1). H2A histone family member Z (H2afz), protein tyrosine phosphatase receptor type C (Ptprc), and serine/arginine-rich splicing factor 2 (Srsf2) were extracted as target genes of rno-miR-107-5p, rno-miR-196b-5p, and rno-miR-3552, respectively. In all cases, there were inverse relationships between the expression patterns of the miRNAs and associated mRNAs: the three miRNAs were all upregulated, and the extracted target mRNAs were all downregulated (Table 4).

To investigate the possibility of applying these miRNAs as human stroke biomarkers, human miRNAs that correspond to these rat miRNAs were identified. Using the database miRBase, human miRNAs with sequences similar to the significantly regulated miRNAs were extracted. All rat miRNAs except one had corresponding human RNAs, and one miRNA pair, hsa-miR-196b-5p and rno-miR-196-5p, had identical sequences (Table 5).

## 3. Discussion

### 3.1. Immunodepression after Stroke

In this study, we conducted a comprehensive gene expression analysis to assess the effect of infarction. The results revealing the downregulation of immune-related pathways (Table 2) and CD molecules (Table 3) in blood indicated that immunosuppression occurred after stroke. This is consistent with previous studies on the alteration of lymphocytes: it was previously reported that the numbers of B cells, T cells, and NK cells in blood decline in MCAO-treated mice [12]. It was also described that stroke-induced immunodeficiency promotes bacterial infections. Although many other studies investigating the immunodepression after stroke have been reported [2,3], the downregulated pathways found in this study may be key to revealing the mechanisms underlying this phenomenon.

While the analysis of rat models revealed immunodepression, this may also be key to revealing the mechanism of immunodepression in stroke patients. In a clinical context, post-stroke pneumonia is a problem; for example, 30-day mortality in stroke patients with pneumonia is 26.9%, compared with 4.4% in those without it [13]. Post-stroke dysphagia and aspiration pneumonia have been considered to be the causes of post-stroke pneumonia, but only 53.8% of patients with post-stroke pneumonia suffer from dysphagia [14]. Immunodeficiency could also be a cause of post-stroke pneumonia [15,16]. In our study, we identified several pathways that may lead to disorder of the immune system. These pathways are possible targets of medical treatments that could prevent post-stroke pneumonia.

### 3.2. mRNA–miRNA Correlation

miRNAs can be transported through the blood–brain barrier (BBB) and through the blood vessels, regulating genes in remote cells [17]. Generally, miRNAs silence particular mRNAs with matching sequences, so there is an inverse relationship between the expression levels of a mRNA and its associated miRNA [18]. In our study, there were three target genes of the significantly regulated mature miRNAs included among the DEGs extracted by DNA microarray analysis of the blood samples (Table 5). In addition, the expression levels of all three miRNA and mRNA pairs were inversely correlated, namely, downregulation of the miRNAs and upregulation of the mRNAs. Therefore, the miRNAs identified in our experiment may be the cause of alterations in the expression of the mRNAs. For example, Ptprc, one of the mRNAs, is known as an important factor in lymphocyte activation and thymic development [19]. Therefore, the regulatory system between miR-196b-5p and Ptprc may be behind the immunodepression after stroke. This information may be helpful to reveal the general mechanisms that occur after ischemic infarction.

### 3.3. miRNA as Novel Biomarkers

Among the seven miRNAs extracted from rat models, human miRNAs with similar sequences were identified for six of them. Furthermore, miR-196b-5p had the exact same sequence between these two species (Table 5). Since the conservation of miRNA sequences between species is high, these miRNAs have potential as biomarkers for human stroke. Some of the blood miRNAs found in rat models were previously reported in brain, hyperoxia, or stress-related diseases, but not in stroke (Table S2) [20,21,22,23,24,25]. In addition, many of the human miRNAs with similar sequences were reported to be related to several diseases, but none was reported to be related to stroke (Table S3) [26,27,28,29,30,31,32,33,34,35,36].

Although there are many studies reporting potential biomarkers for stroke, none was identical with the miRNAs found in this study. The reason for this might rely on the variability of the study. For example, there are studies conducted using different species: humans and rodents [5,7,8]. Although miRNAs are thought to be consistent between species, this can be a great factor of variability. Even when using MCAO model on rodents, the occlusion process varies, such as permanent MCAO model and transient MCAO model [37]. These variabilities might lead to the difference of phenomenon occurring in the body after stroke, resulting in the variety of miRNAs extracted as potential biomarkers.

Overall, the potential biomarker miRNAs in this study were found by focusing on the phenomenon of blood vessel occlusion. By conducting further studies such as using blood samples from human patients, these miRNAs may be validated as novel biomarkers for stroke.

## 4. Materials and Methods

### 4.1. Animals

Male Sprague–Dawley rats (CLEA Japan Inc., Tokyo, Japan), eight weeks of age, weighing 260–300 g, were used in this study (*n* = 14). The rats had free access to food and water and were housed in a temperature- and humidity-controlled room with a 12-h light/dark cycle.

All procedures involving animals conformed to the guidelines for the proper conduct of animal experiments at The University of Tokyo, and were approved by the Animal Care and Use Committee of The University of Tokyo (permit number: P15-147). All surgeries were performed under isoflurane (Wako Pure Chemical Industries, Ltd., Osaka, Japan) anesthesia, and all efforts were made to minimize suffering.

### 4.2. MCAO for Blood Samples

The rats were divided into two groups, MCAO and sham (MCAO: *n* = 11, sham: *n* = 3), and were subjected to MCAO or sham surgery, respectively. Anesthesia was induced with 5% (*v*/*v*) isoflurane and maintained with 2.5% (*v*/*v*) isoflurane. The left common, internal, and external carotid arteries were exposed through a midline cervical incision, and the common and external carotid arteries were ligated using 4-0 silk sutures. For the MCAO group, silicone rubber-coated 4-0 nylon thread with a round tip was inserted through the left internal carotid artery to occlude the origin of the MCA. For the sham group, the same procedure was followed using uncoated 4-0 nylon thread [38]. Both groups were returned to their home cages with free access to food and water and kept in an incubator maintained at 37.5 °C for 90 min [39]. After that, reperfusion was performed by withdrawing the thread and, 24 h after the reperfusion, the rats were anesthetized and the blood was collected from the heart. The blood samples were stored in tubes with TRIzol LS Reagent (Thermo Fisher Scientific, Waltham, MA, USA) at −80 °C.

### 4.3. Measurement of Infarct Volume

Twenty-four hours after reperfusion, the brain was extracted and cut coronally into 2-mm-thick slices using a brain matrix. These slices were stained with TTC (Sigma-Aldrich, St. Louis, MO, USA) to distinguish the infarct part [40]. Photographs of the stained slices were taken using a COOLPIX S9900 digital camera (Nikon, Tokyo, Japan). Using the image analysis software ImageJ (National Institutes of Health), the infarct area was determined by an investigator blinded to the treatment group, in accordance with a previously reported method [41,42,43]. Since the infarct area tends to swell, the method was used with the intention of removing the effect of edema. The volume was calculated by multiplying the area by the thickness of the slice. For each sample, parameters were calculated as follows:

Nonedema volume = (contralateral hemisphere) × 2;
Edema volume = (ipsilateral hemisphere) − (contralateral hemisphere);
Infarct volume = (contralateral hemisphere) − (noninfarcted part of ipsilateral hemisphere);
Edema index = (edema volume)/(contralateral hemisphere);
Infarct index = (infarct volume)/(nonedema volume).

For the experiment from this point, three samples of MCAO group were selected by the criterion of infarct index (17–21%), which resulted in *n* = 3 for each group.

### 4.4. RNA Isolation

Total RNA was extracted from whole blood using TRIzol LS Reagent, following the manufacturer’s protocol. For the mRNA samples, total RNA was purified with the RNeasy Mini Kit (Qiagen, Hilden, Germany). The concentration and purity were checked using a NanoDrop-1000 spectrophotometer (Thermo Fisher Scientific) and Agilent 2100 Bioanalyzer (Agilent Technologies, Santa Clara, CA, USA). For the miRNA samples, the total RNA was used without purification. The concentration and purity were checked with a NanoDrop-1000 spectrophotometer.

### 4.5. DNA Microarray Analysis

Biotin-labeled cRNA was synthesized using a Genechip 3′ IVT PLUS Reagent Kit (Thermo Fisher Scientific). After fragmentation, each sample was hybridized onto a GeneChip Rat Genome 230 2.0 Array (Thermo Fisher Scientific). The procedure was conducted following the manufacturer’s protocol. Statistical analysis was performed with the statistical software program R (ver. 2.7.2, 3.1.2). The CEL files were normalized using the DFW method (R ver. 2.7.2), and PCA was performed using R ver. 3.1.2. Using the Rank Products method (R ver. 3.1.2), probe sets that met the criterion of FDR < 0.05 were extracted as DEGs. Canonical pathway analysis of the DEGs was performed with Qiagen’s Ingenuity Pathway Analysis (IPA, www.qiagen.com/ingenuity) (Qiagen). Pathways with *p*-value < 0.05 and |*Z*-score| ≥ 2 were extracted as being significantly regulated. The microarray data discussed in this report have been deposited in the NCBI’s Gene Expression Omnibus database, and are accessible through GEO Series accession numbers GSE97532 and GSE97533 (https://www.ncbi.nlm.nih.gov/geo/query/acc.cgi?acc=GSE97532, https://www.ncbi.nlm.nih.gov/geo/query/acc.cgi?acc=GSE97533).

### 4.6. miRNA Microarray Analysis

Biotin-labeled RNA was synthesized using a FlashTag Biotin HSR RNA Labelling Kit (Thermo Fisher Scientific). Each sample was hybridized onto a GeneChip miRNA 4.0 Array (Thermo Fisher Scientific), following the manufacturer’s protocol. CEL files were converted to CHP files with the Affymetrix Expression Console software (version 1.4.1, Thermo Fisher Scientific). Statistical analysis was performed by Affymetrix TAC software (version 3.1, Thermo Fisher Scientific) and the genes that met the criteria (ANOVA *p*-value < 0.05 and |fold-change| ≥ 1.5) were extracted as significantly regulated miRNAs. Using the database miRDB (http://mirdb.org), target genes for the miRNAs with a target score ≥80 were extracted [44]. In addition, by using the database miRBase (http://www.mirbase.org, release 21), human miRNAs with sequences similar to the significantly regulated miRNAs were extracted.

## Figures and Tables

**Table 1:** Image analysis of middle cerebral artery occlusion (MCAO)- or sham-treated brain slices.

	Total Volume (mm^3^)	Nonedema Volume (mm^3^)	Edema Volume (mm^3^)	Infarct Volume (mm^3^)	Edema Index (%)	Infarct Index (%)
MCAO						
M1	1326.83	1177.82	149.00	215.50	25.30	18.30
M2	1394.82	1227.18	167.63	255.50	27.32	20.82
M3	1503.44	1281.54	221.90	226.85	34.63	17.70
Mean	1408.36	1228.85	179.51	232.62	29.08	18.94
Sham						
S1	1319.66	1254.14	65.51	0.00	10.45	0.00
S2	1324.36	1289.78	34.58	0.00	5.36	0.00
S3	1275.43	1243.52	31.91	0.00	5.13	0.00
Mean	1306.48	1262.48	44.00	0.00	6.98	0.00

Result of image analysis and measurement of infarct volume. Each parameter is calculated as follows: nonedema volume = (contralateral hemisphere) × 2; edema volume = (ipsilateral hemisphere) − (contralateral hemisphere); infarct volume = (contralateral hemisphere) − (noninfarcted part of ipsilateral hemisphere); edema index = (edema volume)/(contralateral hemisphere); infarct index = (infarct volume)/(nonedema volume).

**Table 2:** Significantly regulated pathways in blood derived from differentially expressed genes.

Ingenuity Canonical Pathways	−log(*p*-Value)	*Z*-Score	Molecules
PI3K signaling in B lymphocytes	2.00	2.00	Cr2, Chp1, Prkcb, Ptprc, Akt2
CD28 signaling in T helper cells	4.41	−2.00	Arpc2, Cd3g, Rt1-Ba, Rt1-Db1, Chp1, Ptprc, Akt2, Rt1-Da
Role of NFAT in regulation of the immune response	3.20	−2.00	Cd3g, Rt1-Ba, Rt1-Db1, Gna12, Chp1, Gng12, Akt2, Rt1-Da
eNOS signaling	2.18	−2.00	Hspa1a, Hspa8, Prkcb, Hsp90ab1, Hsp90aa1, Akt2
Sphingosine-1-phosphate signaling	2.17	−2.00	Rhoh, Gna12, Acer2, Akt2, S1pr1
fMLP signaling in neutrophils	2.07	−2.00	Arpc2, Chp1, Gng12, Prkcb, Cybb
CXCR4 signaling	2.04	−2.00	Rhph, Gna12, Myl12b, Gng12, Prkcb, Akt2
G beta gamma signaling	1.64	−2.00	Gna12, Gng12, Prkcb, Akt2
Fcγ receptor-mediated phagocytosis in macrophages and monocytes	1.57	−2.00	Arpc2, Vamp3, Prkcb, Akt2
Gαq signaling	1.51	−2.00	Rhoh, Chp1, Gng12, Prkcb, Akt2
Signaling by rho family GTPases	1.32	−2.00	Arpc2, Rhoh, Gna12, Myl12b, Gng12, Cybb
Integrin signaling	2.01	−2.24	Arpc2, Rhoh, Itgal, Pfn1, Myl12b, Ppp1cb, Akt2
IL-8 signaling	2.88	−2.65	Rhoh, Irak1, Cr2, Gna12, Gng12, Prkcb, Cybb, Akt2

Pathways with *p*-value < 0.05 and |*Z*-score| ≥ 2, extracted by canonical pathway analysis using IPA. *Z*-score ≥ 2 indicates upregulation and *Z*-score ≤ −2 indicates downregulation in the MCAO group compared with the sham group. Molecules indicate the DEGs included in the pathway (Table S1).

**Table 3:** CD molecules found among the differentially expressed genes from blood samples.

Gene Symbol	Gene Name	Probe Set ID	FDR (M < S)
Cd36	CD36 molecule (thrombospondin receptor)	1367689_a_at	0.0212
		1386901_at	0.0357
Cd3g	CD3 molecule, gamma	1384787_at	0.0466
Cd47	Cd47 molecule	1369559_a_at	0.0192
Cd59	CD59 molecule, complement regulatory protein	1367929_at	0.0186
Cd74	Cd74 molecule, major histocompatibility complex, class II invariant chain	1367679_at	0.0003

Immune-related CD molecules included among the DEGs from blood. FDR (M < S) < 0.05 indicates downregulation in the MCAO group compared with the sham group.

**Table 4:** Significantly regulated miRNAs in blood.

	Signal					
miRNA	MCAO	Sham	Fold Change	*p*-Value	Target Gene	Target mRNA
miR-107-5p	1.08	0.19	1.85	0.022	69	H2afz (M < S)
miR-383-5p	4.26	3.39	1.83	0.024	19	
miR-24-1-5p	2.58	1.91	1.59	0.031	21	
mir-191b	1.06	0.41	1.57	0.004		
miR-196b-5p	1.51	0.90	1.53	0.050	22	Ptprc (M < S)
miR-3552	0.98	0.37	1.52	0.002	74	Srsf2 (M < S)
mir-194-1	0.20	0.99	−1.72	0.017		

miRNAs within ANOVA *p*-value < 0.05 and |fold change| ≥ 1.5. Signal column shows the log2 value of the mean signal of samples from each group. Fold change ≥ 1.5 indicates upregulation and fold change ≤ −1.5 indicates downregulation in the MCAO group compared with the sham group. Target gene column shows the number of target genes of the mature miRNAs from the database miRDB. Target mRNA column shows the target genes that coincide with the DEGs extracted by DNA microarray analysis of the blood sample. M < S indicates that the mRNA is downregulated in the MCAO group compared with the sham group.

**Table 5:** Human miRNAs with sequences similar to significantly regulated miRNAs found in rat blood samples.

Rat miRNA	Sequences	Human miRNA	Sequences	Mismatch
miR-107-5p	24, 1–23	hsa-miR-103a-2-5p	23, 1–23	1
rno-miR-383-5p	21, 2–21	hsa-miR-383-5p	22, 1–20	1
rno-miR-24-1-5p	22, 2–22	hsa-miR-24-1-5p	22, 1–21	0
	22, 2–18	hsa-miR-24-2-5p	22, 1–17	1
rno-mir-191b	120, 15–104	hsa-mir-191	92, 2–91	30
	120, 22–84	hsa-mir-4653	83, 15–77	25
rno-miR-196b-5p	22, 1–22	hsa-miR-196b-5p	22, 1–22	0
	22, 1–22	hsa-miR-196a-5p	22, 1–22	1
rno-miR-3552	21			
rno-mir-194-1	83, 1–83	hsa-mir-194-1	85, 1–84	12
	83, 11–67	hsa-mir-194-2	85, 11–68	10
	83, 60–78	hsa-mir-548az	95, 39–57	1

The human miRNAs were extracted using the database miRBase. The numbers in the “Sequences” columns indicate miRNA length and the position of the sequence aligned between the corresponding miRNAs (start–end). The “Mismatch” column shows the number of different bases included in the aligned sequences.

## Supplementary Materials

Supplementary materials can be found at www.mdpi.com/1422-0067/18/11/2335/s1.
